# Measuring life satisfaction of self-help groups: Role of perceived social support and social empowerment

**DOI:** 10.12688/f1000research.148209.1

**Published:** 2025-01-17

**Authors:** Suraksha Suvarna, Satish Kumar, Navin Kumar Koodamara

**Affiliations:** 1 Department of Commerce, Manipal Academy of Higher Education, Manipal, Karnataka, 576104, India; 2 Department of Commerce, Manipal Academy of Higher Education, Manipal, Karnataka, 576104, India; 3 Manipal Institute of Management, Manipal Academy of Higher Education, Manipal, Karnataka, 576104, India

**Keywords:** Decision Making; External Communication; Life Satisfaction; Perceived Social Support; Social Empowerment; Women Empowerment.

## Abstract

**Background:**

This study was designed to investigate the mediation effects of social empowerment (SE) in the relationship between perceived social support (PSS) and life satisfaction (LS) in women self-help group members. Also, this research attempted to understand the moderating effect of decision making (DM) and external communication (EC) on the relationship between the constructs.

**Methods:**

To achieve the above objective, the primary data were collected from the self-help group women members by using an existing scale. In this survey, 333 participants who are members of self-help group completed the questionnaire and considered for the study. The study is non-experimental and survey-based, with no interventions or manipulations involved. In line with ethical guidelines, we obtained informed consent directly from each respondent before their participation.

**Conclusion:**

The path coefficient values, t-statistics and P-Values confirmed the positive relationship between PSS->LS; PSS->SE & SE->LS in women self-group members. PLS structural equation modelling estimated by the bootstrap method revealed that SE partially mediates the relationship between PSS & LS. With regard to the interaction effect, the slope analysis and f ^2^ effect size confirmed the moderating effect of EC in the relationship between PSS -> LS & SE -> LS.

## 1. Introduction

New and innovative therapeutic modalities, social services, and self-help groups have drawn much research attention in recent years. Research has also examined the outcomes of self-help group participation. One of the most critical themes in self-help group research is women’s social empowerment ( Cheung, Mok, & Cheung, 2005) and satisfaction with life. Women’s social empowerment is widely acknowledged as a critical driver of socioeconomic development, making it a key component of global efforts to promote progress and prosperity. Women’s social empowerment and life satisfaction have received considerable attention because of their potential to provide equal opportunities and outcomes for women, address gender inequities, alleviate poverty, and promote inclusive economic growth. Their contributions and empowerment have been instrumental in driving positive economic growth, and essential drivers of GDP growth ( Chatterjee, Gupta, & Upadhyaya, 2018; Khursheed, Azim Khan, & Mustafa, 2021).

Further, Women’s self-help groups are essential for addressing poverty among marginalized people in developing countries. These organizations support viable socioeconomic growth and empower communities to raise themselves economically by providing financial services to people with little to no income Self-help groups support sustainable growth and offer possibilities for those previously excluded from formal banking (Khursheed, Azim Khan, & Mustafa, 2021).

However, in almost all cultural, social, and political affairs, women face discrimination from society and their families. Females are mistreated and face many obstacles and difficulties in their daily life, which factors restricting women’s empowerment is lack of family support ( Qureshi & Shaikh, 2007). The social status of women consistently affects their life satisfaction ( Kyungeh, Ji-Young, O’Connor, & Wexler, 2008), where they draw less satisfaction from having used borrowed money but gain more satisfaction from joining self-help groups ( Hossain, Asadullah, & Kambhampati, 2019). This means that social status and empowerment are positively related to life satisfaction ( Kyungeh, Ji-Young, O’connor, & Wexler, 2008). Self-help groups play a significant role in promoting economic endeavours and enhancing rural women’s engagement in making decisions for their families. SHGs increase women’s participation in household decision making, access to financial and economic resources, social networks, and bargaining power. The significance of women’s active engagement in decision-making, both within families and on a broader national scale, has been recognized as crucial for attaining equality and fostering peace.

Thus, the relationship among perceived social support, women’s social empowerment, and life satisfaction has attracted considerable research interest. Whether women are satisfied with their lives remains debated in the literature ( Hossain, Asadullah, & Kambhampati, 2019). Empowerment is also an instrument for improving life satisfaction of self-help group members ( Cheung, Mok, & Cheung, 2005). However, an in-depth investigation of the impact of empowerment on life satisfaction in overseas societies is an exciting topic for research ( Cheung, Mok, & Cheung, 2005). Furthermore, the research suggests that the influence of women’s empowerment on life satisfaction should be revisited ( Hossain, Asadullah, & Kambhampati, 2019). Although one study investigated the impact of life satisfaction in Western countries, further study is warranted to determine the differences in women’s life satisfaction in Asian countries ( Kyungeh, Ji-Young, O’connor, & Wexler, 2008). Therefore, this study aims to contribute to the literature on the relationship between perceived social support, women’s social empowerment, and the life satisfaction of self-help group members. This study also investigated the effect of women’s social empowerment on the relationship between perceived social support and life satisfaction.

Further, the present study investigated the interaction effect of women’s decision-making and external communication in the relationship between perceived social support, social empowerment, and life satisfaction of self-help group members. In this context, this study addressed the following research problem: Does perceived social support influence women’s life satisfaction through social empowerment? Does decision-making power and external communication influence the relationship between perceived social support, social empowerment, and life satisfaction? This is the research question of concern in this study.

## 2. Literature

### 2.1 Perceived social support and women’s social empowerment

Women’s empowerment is a process that results in the movement of position from powerless to powerful. This means making women stronger and more financially, socially, and politically skilled. Women’s empowerment is a multidimensional concept in which women achieve greater control over material, financial, and intellectual resources ( Mohapatra & Sahoo, 2016). Perceived social support refers to an individual’s perception of material, psychological, and overall support from friends, family members, and other members of society whenever required ( Ioannou, Kassianos, & Symeou, 2019). Existing literature clarifies that perceived social support advances the sense of social empowerment in women ( Moradi & Funderburk, 2006). Perceived social support is critical predictor of social empowerment in women. Women’s perceived ability to draw attention to needed help contributes to their empowerment ( Cakir & Guneri, 2011). This study indicates that various types of perceived social support impact women’s empowerment differently ( Barringer, Hunter, Salina, & Jason, 2016). Consequently, positive perceived social support helps women empower resources to cope with multiple stressors and belonging support enhances perceived empowerment among women, creating an environment in which they engage in positive activities ( Barringer, Hunter, Salina, & Jason, 2016). However, there are relatively few empirical findings on the relationship between these two variables among self-help group members.

### 2.2 Women’s social empowerment and life satisfaction

Life satisfaction has become an important issue in recent years for most organizations and governments. Countries such as the UK, Germany, Australia, and the USA spend considerable amounts of money and continuously track life satisfaction over time. The level of empowerment is significantly related to life satisfaction, regardless of income, gender, religion, geographical location, and so on ( Hossain et al., 2019). The perception of empowerment substantiates the sense of control over one’s life, improves self-esteem, and increases life satisfaction ( Cheung et al., 2005). Thus, life satisfaction, self-esteem, and a sense of mastery are the outcomes of social empowerment. The study found that women get more life satisfaction from being a self-help group member than from having a say in production decisions ( Hossain et al., 2019).

### 2.3 Perceived social support and life satisfaction

Self-help group members experience several life challenges that require support from others. The others referred to here were family, friends, and colleagues. It is evident from research that support from family and friends is the strongest determinant of life satisfaction ( Akanni & Oduaran, 2018; Kasprzak, 2010). Social support plays a significant role in managing stress, leading to higher life satisfaction ( Mahanta & Agarwal, 2013). Females reported higher levels of perceived social support from friends than males, but there was no significant difference between males and females in terms of social support from family.

Furthermore, females were found to have higher life satisfaction than males ( Mahanta & Agarwal, 2013). An increase in the extent of attachment leads to an increase in life satisfaction and a decrease in attachment among people, leading to a reduction in life satisfaction ( Shahyad, Besharat, Asadi, Shiralipour, & MirnaderMiri, 2011). Thus, perceived social support is a significant factor that influences life satisfaction ( Lu et al., 2015).

**Hypothesis development**

The study’s hypothesis was developed after the conceptual framework was finalized. The first hypothesis explored the relationship between Perceived Social Support (PSS) and Social Empowerment (SE).

H1: Perceived Social Support (PSS) significantly influences Social Empowerment (SE).

The second hypothesis investigates the relationship between perceived social support and life satisfaction.

H2: Perceived Social Support (PSS) positively associated with Life Satisfaction (LS).

hypothesis is developed to understand the nature of the relationship between social empowerment and life satisfaction.

H3: Social Empowerment (SE) is positively related to Life Satisfaction (LS).

The mediating role of SE

The latent construct PSS is hypothesized to influence SE and Effects. The potential mediating effect of social empowerment is interesting to fill this research gap. Therefore, the fourth and fifth hypotheses were developed as follows:

H4: Social Empowerment (SE) significantly mediates the relationship between PSS and LS.

Moderating role of Decision Making (DM) and External Communication (EC)

This study is also interested in understanding and confirming the moderating role of DM and EC in the relationship between the constructs. Hence, this study develops the following hypothesis to test this moderating effect:

H5a: Decision making (DM) significantly moderates the relationship between PSS and LS.

H5b: The DM significantly moderates the relationship between SE and LS.

H6a: EC positively moderates the positive relationship between PSS and LS.

H6b: EC negatively moderates the positive relationship between SE and LS.

H6c: EC negatively moderates the positive relationship between PSS and SE.

## 3. Materials and methods

### 3.1 Sample

In order to assess the hypothesis of the study, a comprehensive empirical investigation was carried out involving the members of three notable self-help groups: the Navodaya Self-Help Group, the Sthri Shakthi Self-Help Group, and the Shri Kshetra Dharmasthala Rural Development (SKDRD) self-help group, all located in the vibrant region of Southern Karnataka, India. The research utilized a judgmental sampling method, a strategic approach that allowed us to select participants based on our informed knowledge and insights. This method was deliberately chosen to ensure that we could target a specific demographic with anticipated characteristics that aligned with the objectives of our study. The final sample comprised a total of 333 individuals.

Among the respondents, the largest segment, accounting for 43%, belonged to the middle age group of 40 to 50 years, indicating a robust representation of middle-aged individuals. In addition, 22% of the participants were in the 50 to 60 age range, while 18% were younger than 30. The study also captured insights from 17% of respondents who were aged 60 and over, highlighting a diverse age spectrum within the groups. More than 63% of the respondents were married, demonstrating a strong commitment to personal relationships, contrasting with approximately 37% who identified as single, divorced, or widowed.

When examining employment status, a significant portion of the respondents, 46%, identified themselves as homemakers, reflecting the traditional roles often observed in the community. Meanwhile, 30% of the population were daily wage earners, who typically engage in various manual labor jobs to support their families. Around 24% were employed in the private or public sector, contributing to the local economy in different professional capacities.

Remarkably, the participants in this study exhibited a deep-rooted connection to their self-help groups, with over 80% having engaged in their activities for more than a year. This statistic underscores not only their commitment but also the meaningful experiences and support that self-help groups provide to their members, fostering personal growth and community resilience.

### 3.2 Data collection procedure

Data were gathered through a carefully designed survey questionnaire administered during the regular weekly meetings of the Self-Help Group (SHG) located in the southern Karnataka. The investigator actively facilitated the survey process, ensuring that all participants understood the questions and had the necessary support to complete the questionnaire. The researcher secured both verbal and written permission from all participants. The informed written consent form was included with the questionnaire. Verbal consent was obtained to affirm understanding of the research and participation in the study. Furthermore, data collection was voluntary, and all responses were anonymized. Participants were informed of their right to withdraw from the survey at any time, with the assurance that their personal information would be kept confidential. To ensure a comfortable setting, each member was allocated 15 minutes to complete the survey at their own pace thoughtfully. Once the members finished, the investigator collected the completed questionnaires.

### 3.3 Measurements

The model formulated in this study comprises five distinct variables that interact to explore the relationship between perceived social support and life satisfaction. The independent variable, perceived social support (PSS), is at the forefront, quantified using a comprehensive 12-item scale developed by Zimet, Dahlem, Zimet, and Farley (1988). This scale assesses the extent to which individuals feel supported by their social networks. The dependent variable under investigation is life satisfaction, evaluated through a 5-item scale created by Diener, Emmons, Larsen, and Griffin (1985). This scale captures individuals’ overall contentment with their lives, providing insights into their subjective well-being.

In addition to these primary variables, the model incorporates one intervening variable, Women’s Social Empowerment, which reflects how women feel empowered to make decisions and take action. Furthermore, the model features two moderators: decision-making and external communication. These moderators examine how the ability to make choices and communicate with others can influence the relationship between perceived social support and life satisfaction. The measures for Women’s Social Empowerment, decision-making, and external communication are derived from a scale developed by Chatterjee, Gupta, and Upadhyay in their 2018 research titled “Empowering Women and Stimulating Development at the Bottom of the Pyramid through Micro-entrepreneurship.”

To gauge responses for all these constructs, participants completed a 5-point Likert scale survey, where a score of 5 indicates “Strongly Agree,” a score of 4 represents “Agree,” a score of 3 signifies “Neutral,” a score of 2 denotes “Disagree,” and a score of 1 means “Strongly Disagree.” This structured approach to measuring the variables enhances the depth and clarity of the study’s findings. Common Method Bias: To mitigate the risk of common method bias, the questionnaire was designed with the constructs arranged in a random order. This strategy was implemented to discourage respondents from making assumptions about causal relationships between the constructs based on their positioning in the survey. Furthermore, the analysis included a collinearity test, which reported a Variance Inflation Factor (VIF) value of less than 5. This result indicates that concerns regarding common method bias are unlikely to affect the findings of this study, thereby enhancing the validity of the results.

## 4. Results

The outer loading of each item indicates the estimated relationship in the reflective measurement model that determines its contribution to the construct. Each indicator’s acceptable level of outer loading for any reflective model was greater than 0.6 ( Henseler, Ringle, & Sarstedt, 2012). Table 1 shows that the outer loading value of each indicator is above the threshold value of 0.6. Therefore, in the present study, there was no problem with the outer loading of any construct indicator. The indicator reliability is the square of each outer loading, where an indicator reliability of 0.4 or higher is acceptable ( Hulland, 1999). Table 1 shows that all indicators have individual reliability values much larger than the minimum adequate level of 0.4.

**Table 1.** Result summary of reflective outer models.

Construct	Indicators	Outer Loadings	Indicator Reliability	Composite Reliability	Convergent Validity AVE	Discriminant validity	VIF
Perceived Social Support	PSS1	0.909	0.826	0.963	0.676	0.822	3.931
	PSS2	0.896	0.802				1.995
	PSS3	0.717	0.514				1.364
	PSS4	0.734	0.538				1.931
	PSS5	0.871	0.758				2.533
	PSS6	0.828	0.685				2.517
	PSS7	0.915	0.837				1.434
	PSS8	0.812	0.659				3.754
	PSS9	0.864	0.746				1.529
	PSS10	0.892	0.795				4.331
	PSS11	0.588	0.345				4.003
	PSS12	0.780	0.608				3.983
Social Empowerment	SWE1	0.875	0.765	0.893	0.691	0.831	2.216
	SWE2	0.916	0.839				3.012
	SWE3	0.711	0.505				1.549
	SWE4	0.808	0.652				1.948
Life Satisfaction	LS1	0.900	0.810	0.918	0.751	0.866	4.559
	LS2	0.902	0.813				4.568
	LS3	0.858	0.736				2.606
	LS4	0.847	0.717				2.474
	LS5	0.822	0.675				2.348
External Communication	EXTWE11	0.711	0.505	0.715	0.670	0.780	3.433
	EXTWE12	0.690	0.476				2.510
	EXTWE13	0.673	0.452				3.434
	EXTWE10	0.754	0.568				3.754
	EXTWE5	0.671	0.450				1.519
	EXTWE6	0.846	0.715				1.331
	EXTWE7	0.881	0.776				1.258
	EXTWE8	0.693	0.480				3.211
	EXTWE9	0.815	0.664				1.960
Decision-Making Power	DWE14	0.899	0.808	0.780	0.710	0.810	3.754
	DWE15	0.956	0.913				4.529
	DWE16	0.922	0.850				2.331

Furthermore, composite reliability or construct reliability measures the internal consistency of each item in the construct. To complete the examination of the structural model, it is essential to establish the reliability and validity of the latent variables. A composite reliability value of 0.7 or more is considered acceptable ( Bagozzi & Yi, 1988). Table 1 describes the composite reliability of all constructs as greater than the threshold level of 0.7. The convergent validity of each latent variable was assessed by examining the Average Variance Extracted (AVE). Generally, an AVE value of 0.5 or above is considered acceptable ( Bagozzi & Yi, 1988). The table indicates that all latent variables have an AVE value exceeding the 0.5 threshold, confirming their convergent validity. To establish discriminant validity, Fornell and Larcker (1981) recommended comparing the square root of AVE for each latent variable with its correlations with other latent variables. If the square root of AVE is greater than the correlations, discriminant validity is achieved. In this study, the square root of AVE for each construct surpassed the correlation values with other constructs, confirming discriminant validity. Additionally, we assessed potential collinearity issues within the inner model by examining the Variance Inflation Factor (VIF). A VIF value below 5 (or a tolerance level above 0.2) is generally considered acceptable to avoid collinearity problems ( Hair, Ringle, & Sarstedt, 2011). Table 1 shows that all VIF values for the variables are below 5, indicating no collinearity issues in the structural model of this study.

### 4.1 Measurement model

**Figure 1.**
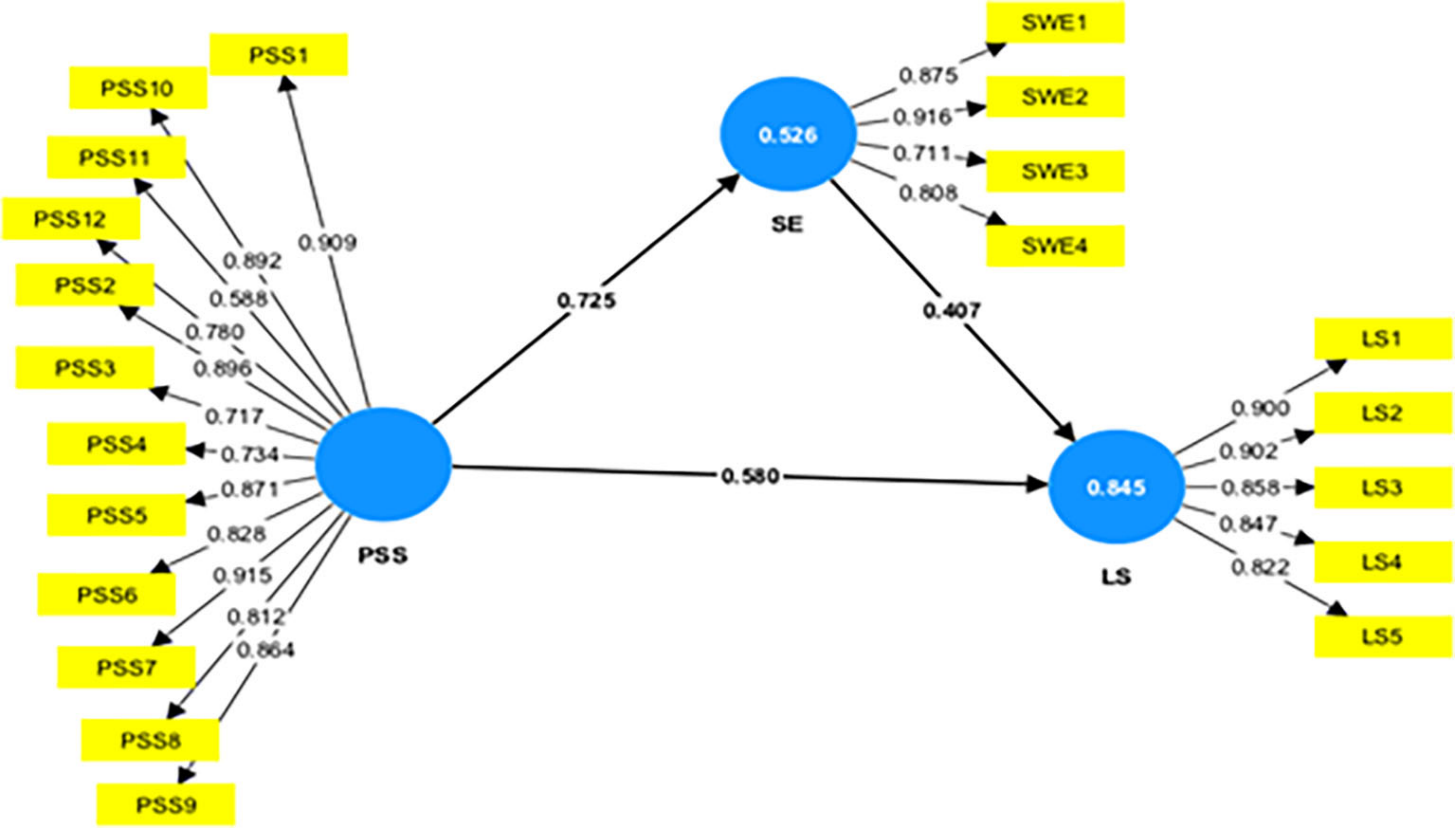
Measurement model to explain the relationship between the variables. Alt Text: Image of the measurement model, which explains the relationship between the PSS, SE, and LS. This image also explains the R ^2^ value (value in the circle) to show the overall impact of the independent variable on the dependent variable.

In Figure 1, the numbers on the arrows are path coefficients that indicate the direct effect of one variable (cause) on another variable (effect). The weights of the different path coefficients enabled us to rank their relative statistical importance. The path coefficient values between the two constructs of the structural model should be more than 0.20 to demonstrate its significance ( Wong, 2013). The path coefficient value in Figure 1 (inner model) explains that Perceived Social Support (PSS) has the most substantial effect on Social Empowerment (0.725) and Life Satisfaction (0.580). Social empowerment was a good predictor of life satisfaction (0.407).

The R ^2^ values or numbers in the circle show the extent to which the other latent variables explain the latent variable’s variance. In management research, the R ^2^ of 0.75 was substantial, 0.50 is moderate, and 0.25 is weak ( Wong, 2013). Figure 1 above shows that the coefficient of determination R ^2^ is 0.845 for the life satisfaction endogenous latent variable, implying that exogenous latent variables, i.e., perceived social support and social empowerment) explain 84.5% of the variance in life satisfaction of women’s self-help group members. Also, Figure 1 illustrates that the coefficient of determination R ^2^ is 0.526 for the women’s social empowerment, implying that perceived social support explains 52.6% variance in the social empowerment (SE).

Table 2 presents the path, t-value, and statistical significance values for all the variables considered for the study. The positive path coefficient value in the relationship between different constructs considered for the study indicates a direct relationship between the two constructs. Using the bootstrapping procedure in the PLS-SEM, T-statistics were checked to see if the inner model’s path coefficient was significant. The path coefficient will be substantial if the T-statistics is higher than 1.96 using a two-tailed T-test with a significance level of 5% ( Wong, 2013). Table 2 explains that PSS (β=0.725, t=30.548, p=0.000) strongly influences the SE, supporting H1. Further, the exogenous variable PSS (β=0.580, t=13.781, p=0.000) and SE (β=0.407, t=9.446, p=0.000) strongly predict the endogenous variable life satisfaction, supporting H2 & H3.

**Table 2.** Path coefficient, T-value and P-values.

Relationship	Path coefficient	SE	T statistics	P values
Perceived Social Support (PSS) and social Empowerment (SE)	0.725	0.024	30.548	0.000
Perceived Social Support (PSS) & Life Satisfaction (LS)	0.580	0.042	13.781	0.000
Social Empowerment (SE) & Life Satisfaction (LS)	0.407	0.043	9.446	0.000

Note: SE=Standard Error.

### 4.2 Mediating analysis

A mediation analysis was performed to assess the mediating role of social empowerment in the relationship between PSS and LS. The results (see Table 3) reveal a significant indirect effect of PSS on life satisfaction (β=0.295, t=9.084, p=0.000). PSS’s total effect of PSS ( Table 3) on LS was significant (β=0.875, t=64.127, p=0.000). However, as shown in Table 3, the effect of PSS on LS was significantly reduced with inclusion of the mediator (SE) (β=0.295, t=9.084, p=0.000). The difference in the values can be easily seen in the results. This shows the mediating role of SE in the relationship between PSS and LS, thus supporting H4.

**Table 3.** Mediating analysis.

Relationship	Original sample	Standard deviation	T statistics	P values
PSS -> SE -> LS	0.295	0.032	9.084	0.000
PSS -> LS	0.875	0.014	64.127	0.000
SE -> LS	0.407	0.043	9.446	0.000

### 4.3 Moderation analysis

This study assessed the moderating role of DM on the relationship between PSS→ LS and SE→ LS. Without including the moderating effect (DM*PSS), the R-squared value for LS is 0.845. This shows that PSS accounts for an 84.5% change in LS. Including the interactive term, the R-square increased to 87.4%. This indicates an increase of 2.9% in variance explained by the dependent variable (LS).

Furthermore, the significance of this moderating effect was analyzed. The results indicate a negative and insignificant moderating impact of DM on the relationship between PSS and LS (β=-0.001, t=0.025, p=0.980), not supporting H5a. This shows that, with an increase in DM, the relationship between PSS and LS is not weakened. Table 4 presents a summary of the moderation analyses. A similar observation was made in the moderating effect of DM on the relationship between SE and LS, not supporting H5b.

**Table 4.** Moderation analysis (decision making).

Relationship	Beta	*SE	T Value	P Value
DM x PSS->LS	-0.001	0.034	0.025	0.980
DM ->LS	0.223	0.025	8.846	0.000
PSS->LS	0.457	0.040	11.465	0.000
DM x SE->LS	-0.031	0.036	0.861	0.389
SE->LS	0.371	0.033	11.279	0.000

Note: SE=Standard Error.

Further, a slope analysis is presented to better understand the nature of the moderating effects ( Figure 3).

**Figure 2.**
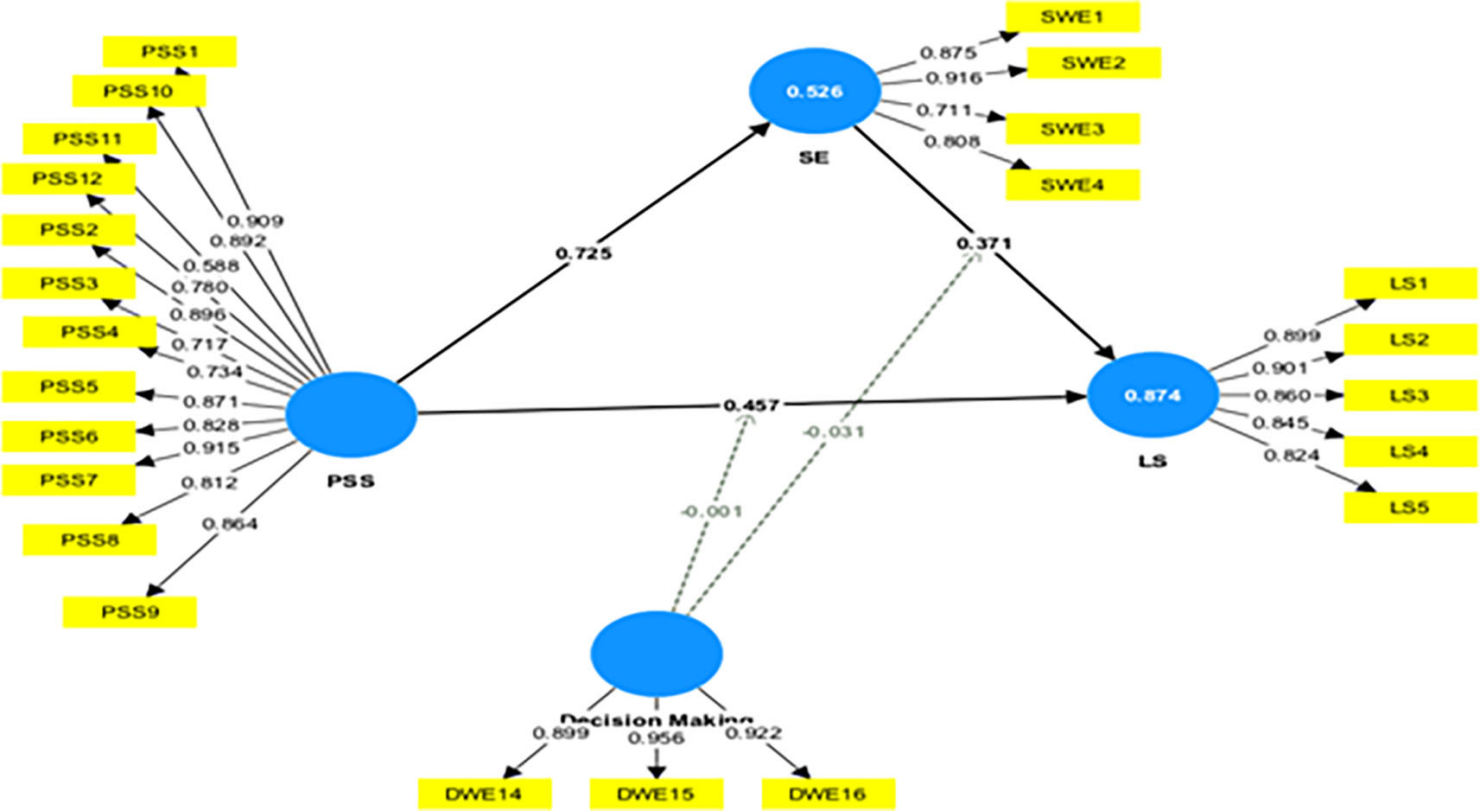
Moderation effect of decision-making. Alt Text: This image shows the moderating effect of decision-making in the relationship between PSS-LS & SE-LS.

As shown in Figure 2, the line is flatter for low DM. This indicates that, at a low level of DM, the impact of PSS on LS is like that of higher DM. At higher DM, the line tends to flatter; this shows that at higher DM, the increase in PSS does not lead to a similar change in LS. In conclusion, a higher DM does not weaken the impact of PSS on LS.

**Figure 3.**
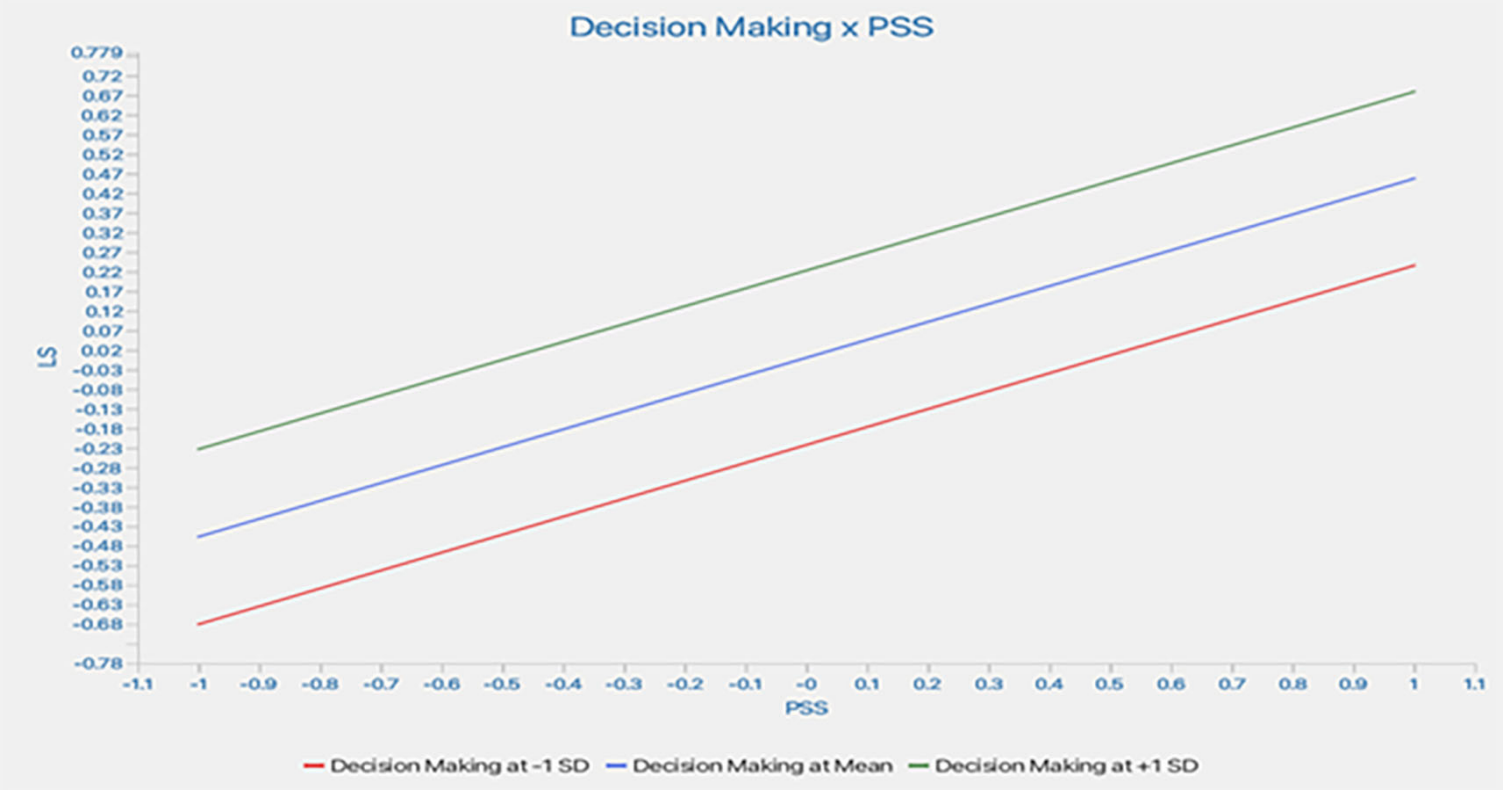
Slope analysis. Alt Text: This image explains the moderating effect of decision-making through the Slope Analysis (Graph). The X-axis represents PSS, and the Y-axis shows the LS.

***F ^2^ Effect size***

Regarding the interactive effect, the f ^2^ effect size indicates the extent to which moderation explains the endogenous construct. The f-square effect size was 0.00, which was insignificant. According to Cohen ( Cohen, 1988), f ^2^ effect size values of 0.02, 0.15, and 0.35 are considered as small, medium, and large effect sizes of moderation, respectively. In this study, there was a negligible or insignificant moderating effect. This indicates that the moderating effect does not contribute significantly to explaining the endogenous construct (LS).

This study assessed the moderating role of EC on the relationship between constructs. Without including the moderating effect (EC*PSS), the R-squared value for LS is 0.845. This shows that PSS accounts for an 84.5% change in LS. Including the interactive term, the R-square increased to 89.9%. This indicates an increase of 5.4% in variance explained by the dependent variable (LS).

**Figure 4.**
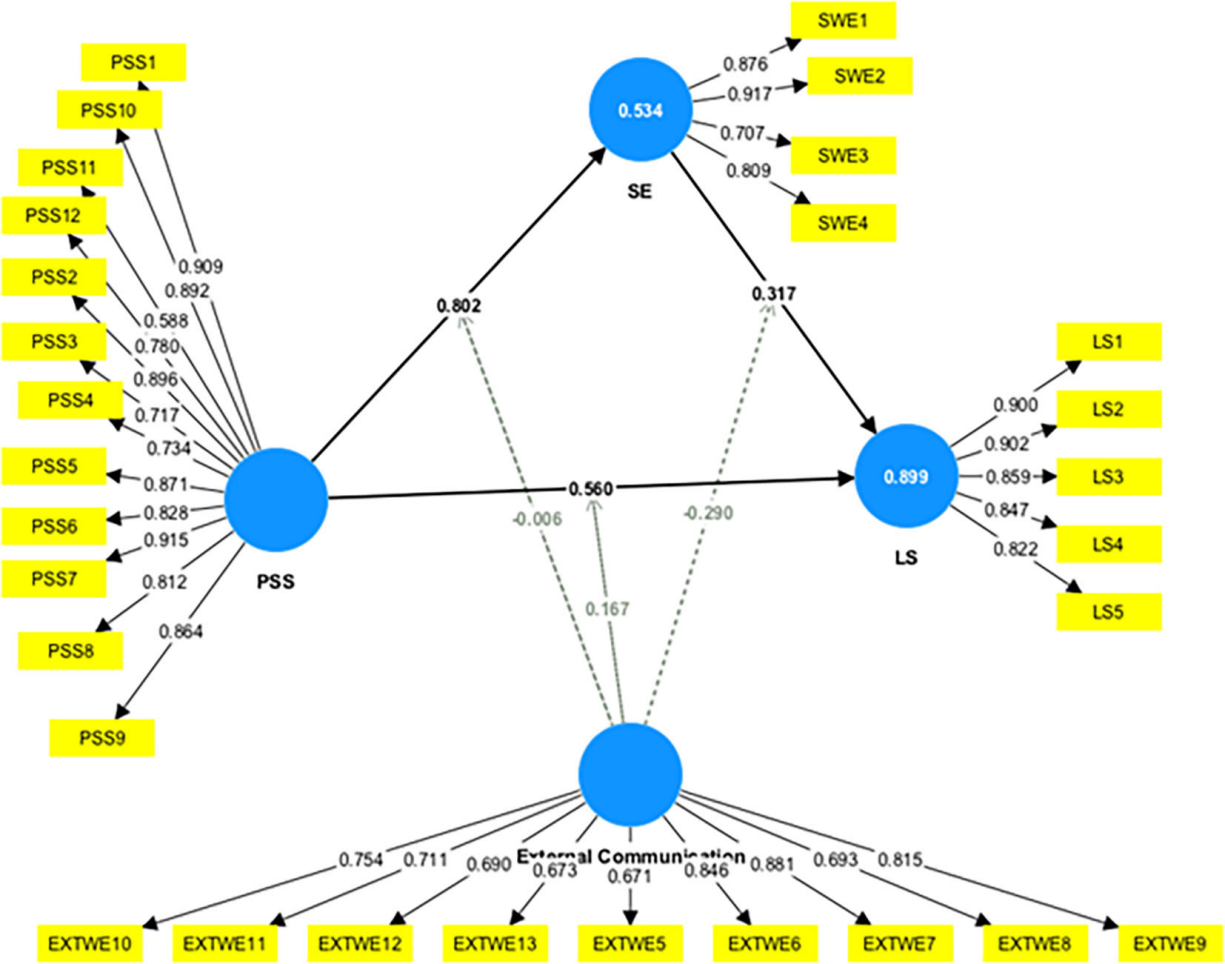
Moderating effect of external communication. Alt Text: This image describes the moderating effect of external communication in the relationship between PSS-LS, PSS-SE, and SE-LS.

Furthermore, the significance of this moderating effect was analyzed as shown in Figure 4. The results indicate a positive and significant moderating impact of EC on the relationship between PSS and LS (β=-0.167, t=5.370, p=0.000), supporting H6a. This indicates that increased EC strengthens the relationship between PSS and LS. Table 5 presents a summary of the moderation analyses.

**Table 5.** Moderating effect of External Communication (EC).

Relationship	Beta	*SE	T Value	P Value
EC x PSS->LS	0.167	0.031	5.370	0.000
EC x SE->LS	-0.290	0.034	8.589	0.000
EC x PSS->SE	-0.006	0.054	0.118	0.906
EC ->LS	0.091	0.046	1.994	0.046
SE->LS	0.317	0.037	8.542	0.000

SE=Standard Error.

**Figure 5.**
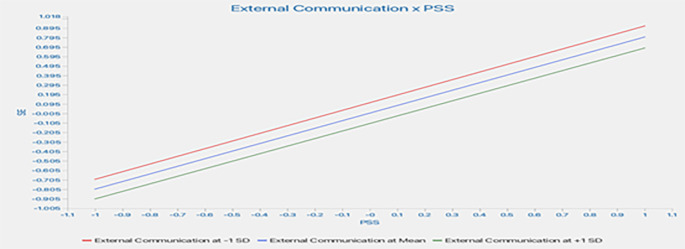
Slope analysis (moderating effect of EC). Alt Text: This image explains the moderating effect of external communication through the Slope Analysis (Graph). The X-axis represents PSS, and the Y-axis shows the LS.

Further, slope analysis is presented to better understand the nature of the moderating effects ( Figure 5).

As shown in Figure 5, the line is much steeper for low EC, indicating that at a low EC level, the impact of PSS on LS is much more robust compared to higher EC. However, at higher EC, the line tends to straighten, indicating that the increase in PSS leads to a similar change in LS at higher EC. In conclusion, higher EC strengthens the impact of PSS on LS.

***F ^2^ Effect size***

Concerning the interactive effect, the f ^2^ effect size indicates the extent to which moderation explains the endogenous construct. The F-squared effect size was 0.136, which was significant. According to Cohen ( Cohen, 1988), the f ^2^ effect size values of 0.02, 0.15, and 0.35 are considered small, medium, and large effect sizes of moderation, respectively. In this study, there was a moderately significant moderating effect. This shows that the moderating effect significantly explains the endogenous construct (LS).

Furthermore, this study assessed the moderating role of EC on the relationship between SE and LS. The results indicate a negative and significant moderating impact of EC on the relationship between SE and LS (β=-0.290, t=8.589, p=0.000), supporting H6b. This shows that an increase in EC weakened the relationship between SE and LS. The F-squared effect size was 0.488, which was significant. This reveals that the EC has a large, significant moderating effect. This shows that the moderating effect of EC significantly contributes to explaining the endogenous construct (LS). However, the moderating effect of EC on the relationship between PSS and SE was insignificant (β=-0.006, t=0.118, p=0.906), which does not support H6c.

## 5. Discussion

This research contributes to the existing knowledge on the role of perceived social support (PSS) and social empowerment (SE) on life satisfaction (LS) among female self-help group members. This study also describes the mediating effect of SE on the relationship between PSS and LS. Furthermore, this study empirically tested the interaction effect of decision-making (DM) and external communication (EC) on the relationships between PSS, SE, and LS. The present study revealed a significant positive association between PSS-SE, PSS-LS, and SE-LS scores. The results of the current study corresponded with the findings of other research that explains how social support to SHG women members (family, friends, relatives, etc.) empowers her socially ( Ghasemi, Badsar, Falahati, & Karamidehkordi, 2021; Dakhan, Sohu, Mustafa, Asadullah, & Sohu, 2021). The patient’s life satisfaction improved ( Alorani & Alradaydeh, 2018; Danielsen, Samdal, Hetland, & Wold, 2009). This implies that social support substantially affects social empowerment and life satisfaction. Social support changes women’s social status. It is evident from the findings of the present study that social support plays a significant role in women’s empowerment, which is consistent with the findings of previous studies ( Reininger, Pe’rez, Flores, Chen, & Rahbar, 2012; Ghasemi, Badsar, Falahati, & Karamidehkordi, 2021; Dakhan, Sohu, Mustafa, Asadullah, & Sohu, 2021). These findings suggest that women feel empowered when support is provided ( Chou et al., 2018). Support from family or friends also plays a significant role in handling stressful situations and boosting psychological wellbeing.

Moreover, perceived social support provides women with self-help group members with better interpersonal relationships and comfort, leading to better life satisfaction. Thus, it is evident from this study that social support promotes life satisfaction, which is consistent with the results of previous studies ( Akanni & Oduaran, 2018; Alorani & Alradaydeh, 2018; Danielsen, Samdal, Hetland, & Wold, 2009; Shahyad et al., 2011; Ali et al., 2010). These findings signify that women who receive high social support have positive evaluations of their lives. Support may be in the form of advice, counseling, financial assistance, or timely help, which ultimately leads to a sense of life satisfaction. Therefore, women in the self-help group perceive a satisfied life if they provide social support. Family members should encourage women to participate in self-help group-related activities, which improves their social networks and, consequently, their life satisfaction.

Further, this study has shown that life satisfaction is a prominent outcome of the social empowerment of female members of SHG, which is consistent with the findings of other studies ( Cheung, Mok, & Cheung, 2005; Hossain et al., 2019). These findings suggest that family relations, marital relations, and self-confidence improved after joining the self-help groups, which helped boost life satisfaction. This means judging how satisfied women are with their present state of affairs, based on their social empowerment. Therefore, the judgment of the overall life satisfaction of women is based on their sense of improvement in their marital or family relations.

Therefore, this study answered affirmatively to the question: Will SHG members who experience a higher degree of social support and empowerment report a higher degree of life satisfaction? The findings also suggest that the state’s efforts in the last decade to support SHG members have effectively empowered and enhanced life satisfaction.

This is the first study to explore how social support affects SHG life satisfaction using social empowerment as an intervening variable. It was statistically proven that the impact of social support on life satisfaction was passed through social empowerment. Feelings of social support might increase the sense of empowerment of SHG members, thereby improving their life satisfaction. Therefore, the current study’s findings highlight the significance of the context within which women’s social empowerment influences the relationship between social support and life satisfaction. Moreover, this research makes a significant contribution to the existing literature by assessing the moderating effect of decision-making and external communication on the relationship between PSS-LS and SE-LS. The moderating effect of decision making on the relationship between PSS-LS and SE-LS is insignificant. However, the moderating effect of external communication on the relationship between PSS-LS and SE-LS was significant. These results indicate that external communication is an important index that reflects empowerment and life satisfaction. This means that the higher the level of external communication, the greater is the effect of social support on life satisfaction and empowerment.

## 6. Implications

Policymakers need to be aware that social support and empowerment are vital to women’s life satisfaction. Through the self-help group (SHG), policymakers should encourage the role of psychological counselors in conducting an assessment of female members with low social support, low empowerment, and less satisfaction. The state should provide the necessary programs for women’s participation in SHG activities to develop a social network. This helps them to create a plan of action to promote self-help group members’ sense of social support, empowerment, and life satisfaction. Furthermore, SHG managers can empower their members by facilitating solid social support, leading to life satisfaction. It is also essential to encourage and motivate women to participate in external communication, which strengthens the relationship between social support, empowerment, and life satisfaction.

## 7. Limitations and future scope of research

This study was a cross-sectional survey, in which it was difficult to precisely determine and analyze the causal relationships between variables over time. In addition, cross-sectional analyses may be prone to bias. Therefore, longitudinal studies should be considered in the future. Qualitative research may also explain the associations between variables. Further, the current study evaluated a sample from the three significant SHGs, Navodaya, Sthri Shakthi, and Shri Kshetra Dharmasthala Rural Development (SKDRD), ignoring the members of the other SHG are present in these areas. Future research should consider samples from all SHGs to generalize our findings.

## 8. Conclusion

This study concluded that perceived social support and social empowerment are significant in improving the life satisfaction of self-help group members. We have attempted to shed light on social support and empowerment as a mechanism to increase the life satisfaction of female SHG members. The results of the present study further revealed that social empowerment mediates the relationship between perceived social support and life satisfaction, which is a unique finding of this study. Moreover, this study demonstrated for the first time that external communication intensifies the influence of perceived social support and social empowerment on life satisfaction.

Ethics and consent: Given the non-experimental, survey-based design of this research, formal ethical approval was taken from the Institutional ethics committee. The signed informed consent was collected from each research participant. The informed consent form was included with the questionnaire. The researcher secured both verbal and written permission from all participants. The verbal consent was obtained to affirm understanding of the research and participation in the study. Furthermore, data collection was voluntary, and all responses were anonymized. Participants were informed of their right to withdraw from the survey at any time, with assurances that their personal information would be kept confidential.

Approval No: IECI: 412/2022

Date of Approval: 14 ^th^ December 2022

Name: Kasturba Medical College and Kasturba Hospital Institutional Ethics Committee

## Data Availability

Dryad: Coded respondents survey data to measure life satisfaction of self-help group women members. https://doi.org/10.5061/dryad.6hdr7sr9f ( Suvarna *et al.*, 2024). The project contains the following underlying data:
- Response_Sheet.csv Data are available under the terms of the Creative Commons Zero “No rights reserved” data waiver (CC0 1.0 Public domain dedication). Dryad: Coded respondents survey data to measure life satisfaction of self-help group women members. https://doi.org/10.5061/dryad.6hdr7sr9f ( Suvarna *et al.*, 2024). The project contains the following extended data:
- README.md (Questionnaire) Data are available under the terms of the Creative Commons Zero “No rights reserved” data waiver (CC0 1.0 Public domain dedication).
