# Supplementary material for: Measuring life satisfaction of self-help groups: Role of perceived social support and social empowerment
Source: F1000Res. 2025 Mar 6;14:101. Originally published 2025 Jan 17. [Version 2] doi: 10.12688/f1000research.148209.2 (PMC11947657; doi:10.12688/f1000research.148209.2)
Supplement: Peer review history

# Reviewer report: Reviewer response for version 2

**DOI:** 10.5256/f1000research.179008.r371650

**Published:** 2025-07-10

**Authors:** Virginia Barba-Sanchez

**Affiliations:** 1 ENSITMA R.G., Department of Business Science, Universidad de Castilla-La Mancha, Albacete, Spain

Thank you for the opportunity to review the manuscript ‘Measuring life satisfaction of self-help groups: Role of perceived social support and social empowerment’ submitted to F1000Research. I enjoyed reading the manuscript and found it to be an informative study of factors that affect the life satisfaction of self-help groups.

 Overall, the research addresses the relevant topic and takes as a starting point some questions that require further investigation. These research questions should be developed in a theoretical and conceptual framework. Empirical analysis has been carried out to provide evidence for research questions, but their research hypotheses are wrongly formulated, since readers need not know what a mediating and/or moderating effect is. I would highly recommend that the authors rewrite the hypotheses. The results are appropriately interpreted and discussed. So, the manuscript has potential, but the reasoning does not flow naturally. The paper must be improved in line with the comments below:

1. The last three sentences of the abstract are superfluous in the summary; however, it would be good to specify the implications of the study.
2. Regarding the Introduction, the first two paragraphs need to be enhanced with more arguments exemplifying the importance of life satisfaction and this research. There is a lot of published work, such as Barba-Sanchez et al. (2024) (Ref 1), to strengthen the narrative on this.
3. In the introduction, it is very worthwhile to introduce readers in more detail to the social and cultural context in southern Karnataka.
4. Section 2 should integrate the hypotheses. Please rewrite the hypotheses.
5. In section 2, you introduce the theoretical framework, but it is a succession of empirical works without theoretical cohesion. Please explain.
6. The sample has been chosen to demonstrate that it is representative of the society being studied. We would also recommend adding a table with accurate information about the technical specifications of the study and another table with the comparative demographics between the population and sample.
7. Please specify the different items used to compose the different measurements. It could incorporate them in one simplified table, which shows a list of items used to measure each of the hypotheses.
8. Why aren’t control variables used?
9. Please add the Conbrach’s Alpha and the HTMT0.85 criteria in Table 1.
10. Please add an endogeneity study to ensure that no important variables are missing from the model.
11. An article must be self-contained, i.e. it must contain all the information necessary for its comprehension. In this sense, please add the direct effect, t-value, p-value, percentile confidence interval (PCI), and f ^2^ in Table 2. Please add the total effect and indirect effect, t-value, VAF, and percentile confidence interval (PCI) in Table 3. In addition, please add a new table with the moderation effects of decision-making and external communication. Please note that your proposed model corresponds to Hayes's (2015) case number 58.
12. The conclusion and discussion sections lack significant theoretical references to the findings.

We hope you find the above comments useful and we wish you the best of luck with developing the paper further.

Is the work clearly and accurately presented and does it cite the current literature?

Partly

If applicable, is the statistical analysis and its interpretation appropriate?

Partly

Are all the source data underlying the results available to ensure full reproducibility?

Yes

Is the study design appropriate and is the work technically sound?

Partly

Are the conclusions drawn adequately supported by the results?

Yes

Are sufficient details of methods and analysis provided to allow replication by others?

Partly

Reviewer Expertise:

Measuring social value, Entrepreneurship, Structural equations.

I confirm that I have read this submission and believe that I have an appropriate level of expertise to confirm that it is of an acceptable scientific standard, however I have significant reservations, as outlined above.

## References

[1] : How entrepreneurship drives life satisfaction among people with intellectual disabilities (PwID): a mixed-method approach. *Management Decision* .2024;62(2) : 10.1108/MD-11-2022-1568 426-449 10.1108/MD-11-2022-1568

[2] : An Index and Test of Linear Moderated Mediation. *Multivariate Behavioral Research* .2015;50(1) : 10.1080/00273171.2014.962683 1-22 10.1080/00273171.2014.962683 26609740

# Reviewer report: Reviewer response for version 2

**DOI:** 10.5256/f1000research.179008.r371644

**Published:** 2025-04-08

**Authors:** Nikhil Sapre

**Affiliations:** 1 University of Bristol Business School, Bristol, UK

This study examines the life satisfaction of women in self help groups in Southern India. The literature review critically evaluates recent studies leading to six well developed hypotheses. Appropriate and robust empirical methods have been employed. The discussion situates the results in the broader literature and makes sound policy recommendations. Concerns around the sample and research design raised on the first submission, have been sufficiently addressed in the current version.

Is the work clearly and accurately presented and does it cite the current literature?

Yes

If applicable, is the statistical analysis and its interpretation appropriate?

Yes

Are all the source data underlying the results available to ensure full reproducibility?

Yes

Is the study design appropriate and is the work technically sound?

Yes

Are the conclusions drawn adequately supported by the results?

Yes

Are sufficient details of methods and analysis provided to allow replication by others?

Yes

Reviewer Expertise:

Banking, Social Finance

I confirm that I have read this submission and believe that I have an appropriate level of expertise to confirm that it is of an acceptable scientific standard.

# Reviewer report: Reviewer response for version 2

**DOI:** 10.5256/f1000research.179008.r371647

**Published:** 2025-03-26

**Authors:** Navya Gubbi Sateeshchandra

**Affiliations:** 1 Berlin School of Business and Innovation, Berlin, Berlin, Germany

The article highlights how life satisfaction is impacted by a perceived social support and social empowerment. The study is significant in the field of social science and helpful to other researchers. The researchers have made an effort not only to examine the direct relationships but also to explore how mediators influence the connection.

 Perceived social support and social empowerment is an important factor in life satisfaction. The title of the article attracts interested scholars and practitioners. The author has made efforts to address the importance of social support and women empowerment by exploring the complex relationship between social support and life satisfaction. 

 Further, the findings of the study can help policy makers to develop strategies towards women empowerment and improve life satisfaction of self-help group members. The robust methodology ensures an insightful understanding by making the purpose of the study evident and valuable for various practitioners, NGO's and policy makers.

 In this context, I approve this article without any revisions.

Is the work clearly and accurately presented and does it cite the current literature?

Yes

If applicable, is the statistical analysis and its interpretation appropriate?

Yes

Are all the source data underlying the results available to ensure full reproducibility?

Yes

Is the study design appropriate and is the work technically sound?

Yes

Are the conclusions drawn adequately supported by the results?

Yes

Are sufficient details of methods and analysis provided to allow replication by others?

Yes

Reviewer Expertise:

Economics, institutional Economics, Public Policy, Sustainable Development

I confirm that I have read this submission and believe that I have an appropriate level of expertise to confirm that it is of an acceptable scientific standard.

# Reviewer report: Reviewer response for version 2

**DOI:** 10.5256/f1000research.179008.r369751

**Published:** 2025-03-14

**Authors:** Andika Pratama

**Affiliations:** 1 Community Education, Universitas Sriwijaya (Ringgold ID: 175522), Palembang, South Sumatra, Indonesia; 2 Universitas Pendidikan Indonesia, Bandung, Indonesia

Having carefully assessed the revisions made to the manuscript, I am pleased to confirm that the authors have satisfactorily addressed the concerns raised in my previous review. The incorporation of an acknowledgement of social desirability bias within the methodology section, along with a more detailed explanation of why decision-making does not moderate the relationship between social support and life satisfaction, significantly enhances the clarity and robustness of the study. Furthermore, the expanded discussion on the role of external communication and the delineation of barriers to policy intervention provide a more comprehensive understanding of the findings.

 Given these improvements, I find the revised manuscript sufficiently aligned with the required standards and am happy to amend my previous status of *Approved with reservations* to *Approved.*

Is the work clearly and accurately presented and does it cite the current literature?

Partly

If applicable, is the statistical analysis and its interpretation appropriate?

Partly

Are all the source data underlying the results available to ensure full reproducibility?

Yes

Is the study design appropriate and is the work technically sound?

Yes

Are the conclusions drawn adequately supported by the results?

Partly

Are sufficient details of methods and analysis provided to allow replication by others?

Yes

Reviewer Expertise:

I am a researcher and practitioner in the fields of social work, community education, and community empowerment. My expertise enables me to rigorously evaluate scholarly research, ensuring that studies contribute meaningfully to academic discourse

I confirm that I have read this submission and believe that I have an appropriate level of expertise to confirm that it is of an acceptable scientific standard.

# Reviewer report: Reviewer response for version 1

**DOI:** 10.5256/f1000research.162492.r363968

**Published:** 2025-02-27

**Authors:** Andika Pratama

**Affiliations:** 1 Community Education, Universitas Sriwijaya (Ringgold ID: 175522), Palembang, South Sumatra, Indonesia; 2 Universitas Pendidikan Indonesia, Bandung, Indonesia

The article, " ***Measuring Life Satisfaction of Self-Help Groups: Role of Perceived Social Support and Social Empowerment***," presents a relevant and contemporary analysis of the interplay between perceived social support, social empowerment, and life satisfaction among women members of self-help groups. While the study contributes to the existing body of literature on social empowerment and its psychological and social benefits, several critical aspects warrant closer examination.

 The conceptual foundation of the study is well-framed within the broader discourse on women’s empowerment, but certain theoretical gaps are apparent. The literature review, though extensive, lacks a nuanced critique of prior studies, particularly concerning methodological limitations and conflicting findings. While multiple references support the claims made, the integration of theoretical perspectives remains relatively superficial. The discussion on the role of social empowerment in enhancing life satisfaction is compelling but does not sufficiently address alternative explanations or counterarguments that could provide a more balanced understanding.

 The methodology demonstrates a systematic approach, yet there are notable concerns regarding sampling and data collection. The use of judgmental sampling, while justified for targeting specific demographics, raises questions about selection bias and the generalizability of findings. The study states that 333 participants were surveyed from three self-help groups in Southern Karnataka, India, but does not clarify whether these groups are representative of the broader population of self-help group members across different cultural and socioeconomic contexts. The reliance on self-reported measures, though common in social science research, introduces the potential for social desirability bias, which the study does not explicitly acknowledge or control for.

 The statistical analysis is robust, employing partial least squares structural equation modeling (PLS-SEM) with bootstrapping. The authors present clear evidence of mediation effects, reinforcing the claim that social empowerment partially mediates the relationship between perceived social support and life satisfaction. However, the moderation analysis results are less conclusive, with decision-making showing an insignificant moderating effect while external communication exhibits a significant impact. The interpretation of these findings could be expanded to explore why decision-making did not exert a moderating influence, especially given the broader discourse on women’s empowerment and autonomy.

 One notable strength of the study is its focus on external communication as a moderating variable, an aspect often overlooked in similar research. The findings suggest that higher levels of external communication strengthen the relationship between perceived social support and life satisfaction. However, the study could have further interrogated the mechanisms underlying this effect. For instance, does external communication facilitate access to better resources, enhance self-efficacy, or contribute to greater community engagement? Addressing these aspects would enhance the study’s explanatory power.

 The discussion and implications section aligns with the study’s objectives, providing practical recommendations for policymakers, self-help group managers, and social workers. The emphasis on fostering external communication to enhance life satisfaction is particularly valuable. However, the study could benefit from a more critical engagement with policy implications. While it advocates for increased social support initiatives, it does not sufficiently address structural barriers such as gender norms, economic constraints, and institutional limitations that may impede the effectiveness of such interventions.

 Despite its contributions, the study has limitations that should have been more explicitly acknowledged. The cross-sectional design restricts the ability to infer causality, and while the authors suggest longitudinal studies for future research, they do not outline a clear methodological framework for how such studies could be conducted. Additionally, qualitative insights from self-help group members could have provided richer contextual understanding and complemented the quantitative findings.

 Overall, this study makes a meaningful contribution to understanding the dynamics of social support, empowerment, and life satisfaction among women in self-help groups. However, it would benefit from a more critical engagement with theoretical perspectives, a more rigorous discussion of methodological constraints, and a deeper exploration of the mechanisms driving the observed relationships.

Is the work clearly and accurately presented and does it cite the current literature?

Partly

If applicable, is the statistical analysis and its interpretation appropriate?

Partly

Are all the source data underlying the results available to ensure full reproducibility?

Yes

Is the study design appropriate and is the work technically sound?

Yes

Are the conclusions drawn adequately supported by the results?

Partly

Are sufficient details of methods and analysis provided to allow replication by others?

Yes

Reviewer Expertise:

I am a researcher and practitioner in the fields of social work, community education, and community empowerment. My expertise enables me to rigorously evaluate scholarly research, ensuring that studies contribute meaningfully to academic discourse

I confirm that I have read this submission and believe that I have an appropriate level of expertise to confirm that it is of an acceptable scientific standard, however I have significant reservations, as outlined above.

# Response

**Published:** 2025-03-05

**Authors:** SATISH KUMAR

**Affiliations:** Department of Commerce, Manipal Academy of Higher Education, Manipal, Karnataka, India

**Reviewer Comments:** The conceptual foundation of the study is well-framed within the broader discourse on women’s empowerment, but certain theoretical gaps are apparent. The literature review, though extensive, lacks a nuanced critique of prior studies, particularly concerning methodological limitations and conflicting findings. While multiple references support the claims made, the integration of theoretical perspectives remains relatively superficial. The discussion on the role of social empowerment in enhancing life satisfaction is compelling but does not sufficiently address alternative explanations or counterarguments that could provide a more balanced understanding.

 Author Response: Thank you for the valuable suggestions. We have addressed the concern positively in the literature section pertaining to social empowerment and life satisfaction.

 Reviewer Comment: The methodology demonstrates a systematic approach, yet there are notable concerns regarding sampling and data collection. The use of judgmental sampling, while justified for targeting specific demographics, raises questions about selection bias and the generalizability of findings. The study states that 333 participants were surveyed from three self-help groups in Southern Karnataka, India, but does not clarify whether these groups are representative of the broader population of self-help group members across different cultural and socioeconomic contexts. The reliance on self-reported measures, though common in social science research, introduces the potential for social desirability bias, which the study does not explicitly acknowledge or control for.

 Authors Response: Thank you for the comments. We have acknowledged the social desirability bias in the methodology section.

 Reviewer Comment: The statistical analysis is robust, employing partial least squares structural equation modeling (PLS-SEM) with bootstrapping. The authors present clear evidence of mediation effects, reinforcing the claim that social empowerment partially mediates the relationship between perceived social support and life satisfaction. However, the moderation analysis results are less conclusive, with decision-making showing an insignificant moderating effect while external communication exhibits a significant impact. The interpretation of these findings could be expanded to explore why decision-making did not exert a moderating influence, especially given the broader discourse on women’s empowerment and autonomy.

 Authors Response: Thank you for the suggestions. We have responded positively to the concern raised in the discussion section.

 Reviewer Comments: One notable strength of the study is its focus on external communication as a moderating variable, an aspect often overlooked in similar research. The findings suggest that higher levels of external communication strengthen the relationship between perceived social support and life satisfaction. However, the study could have further interrogated the mechanisms underlying this effect. For instance, does external communication facilitate access to better resources, enhance self-efficacy, or contribute to greater community engagement? Addressing these aspects would enhance the study’s explanatory power.

 Author Response: We have addressed this concern in the discussion section.

 The discussion and implications section aligns with the study’s objectives, providing practical recommendations for policymakers, self-help group managers, and social workers. The emphasis on fostering external communication to enhance life satisfaction is particularly valuable. However, the study could benefit from a more critical engagement with policy implications. While it advocates for increased social support initiatives, it does not sufficiently address structural barriers such as gender norms, economic constraints, and institutional limitations that may impede the effectiveness of such interventions.

 Author Response: Thank you for the suggestions. We have addressed this concern positively in the limitations section.

 Reviewer Comments: Despite its contributions, the study has limitations that should have been more explicitly acknowledged. The cross-sectional design restricts the ability to infer causality, and while the authors suggest longitudinal studies for future research, they do not outline a clear methodological framework for how such studies could be conducted. Additionally, qualitative insights from self-help group members could have provided richer contextual understanding and complemented the quantitative findings.

 Author Response: Thank you for the suggestions. We have addressed this concern positively in the limitations section.

 Reviewer Comments: Overall, this study makes a meaningful contribution to understanding the dynamics of social support, empowerment, and life satisfaction among women in self-help groups. However, it would benefit from a more critical engagement with theoretical perspectives, a more rigorous discussion of methodological constraints, and a deeper exploration of the mechanisms driving the observed relationships.

 Author Response: We have incorporated all the changes suggested by the reviewer. Thank you so much for your time and consideration.

# Reviewer report: Reviewer response for version 1

**DOI:** 10.5256/f1000research.162492.r365390

**Published:** 2025-02-18

**Authors:** Josue Turpo-Chaparro

**Affiliations:** 1 Universidad Peruana Union, Lima District, Lima Region, Peru

1. Explain in the introduction with example how self-help groups empower people economically. 

 2. It is important to frame the main theory under which the study is being carried out

 3. When it comes to almost all cultural, social and political contexts, women face discrimination, it could be clearer. Are you specifically describing an Asian context?

 4. It is important that in the introduction or in the literature review you place the concepts of the variables you are using

 5. In the methodology, although 333 people are included, it would be useful to know the total population

 6. When you mention 63% that they were married, does that mean they were legally married or were some couples cohabiting?

 7. In the data collection process, although it is specified that approval was obtained from the ethics committee, it is necessary to specify what the criteria were for excluding minors since it is possible that some women could have participated as minors but legally married.

 8. In relation to the instruments used, clearly state whether these were adapted or translated 

 9. Specify what psychometric properties the instruments used have.

 10. Specify the test of normality of the data, if this was through the Standard Deviation and Kurtosis or if it was through another test

 11. It is necessary to place the practical aspects of the research in the discussion

 12. In the discussion place the theoretical implications

 13. It is important that the sample be placed in the limitation, since the type of sampling used has disadvantages and it is necessary to propose future studies 

 14. Place an additional limitation on self-reports used in research

Is the work clearly and accurately presented and does it cite the current literature?

Partly

If applicable, is the statistical analysis and its interpretation appropriate?

Yes

Are all the source data underlying the results available to ensure full reproducibility?

Yes

Is the study design appropriate and is the work technically sound?

Partly

Are the conclusions drawn adequately supported by the results?

Yes

Are sufficient details of methods and analysis provided to allow replication by others?

Partly

Reviewer Expertise:

Research methodology. Psychometric and bibliometric studies.Organizational psychology

I confirm that I have read this submission and believe that I have an appropriate level of expertise to state that I do not consider it to be of an acceptable scientific standard, for reasons outlined above.
